# Sustainable healthcare – Time for ‘Green Podiatry’

**DOI:** 10.1186/s13047-021-00483-7

**Published:** 2021-06-15

**Authors:** Angela Margaret Evans

**Affiliations:** Discipline of Podiatry, School of Allied Health, Human Services and Sport, La Trobe University, Melbourne, Victoria 3086 Australia

**Keywords:** Climate change, Healthcare, Carbon, Greenhouse, Podiatry, Emissions

## Abstract

**Background:**

Healthcare aims to promote good health and yet demonstrably contributes to climate change, which is purported to be ‘*the biggest global health threat of the 21st century’*. This is happening now, with healthcare as an industry representing 4.4% of global carbon dioxide emissions.

**Main body:**

Climate change promotes health deficits from many angles; however, primarily it is the use of fossil fuels which increases atmospheric carbon dioxide (also nitrous oxide, and methane). These greenhouse gases prevent the earth from cooling, resulting in the higher temperatures and rising sea levels, which then cause ‘wild weather’ patterns, including floods, storms, and droughts.

Particular vulnerability is afforded to those already health compromised (older people, pregnant women, children, wider health co-morbidities) as well as populations closer to equatorial zones, which encompasses many low-and-middle-income-countries. The paradox here, is that poorer nations by spending less on healthcare, have lower carbon emissions from health-related activity, and yet will suffer most from global warming effects, with scant resources to off-set the increasing health care needs.

Global recognition has forged the Paris agreement, the United Nations sustainable developments goals, and the World Health Organisation climate change action plan. It is agreed that most healthcare impact comes from consumption of energy and resources, and the production of greenhouse gases into the environment.

Many professional associations of medicine and allied health professionals are advocating for their members to lead on environmental sustainability; the Australian Podiatry Association is incorporating climate change into its strategic direction.

**Conclusion:**

Podiatrists, as allied health professionals, have wide community engagement, and hence, can model positive environmental practices, which may be effective in changing wider community behaviours, as occurred last century when doctors stopped smoking.

As foot health consumers, our patients are increasingly likely to expect more sustainable practices and products, including ‘green footwear’ options. Green Podiatry, as a part of sustainable healthcare, directs us to be responsible energy and product consumers, and reduce our workplace emissions.

## Background

Climate change is damaging people’s health now, and will have greater impact in the future. Citing the Lancet; “Climate change is the biggest global health threat of the 21st century” [1].

Ironically, health care (medical, allied health, inpatient, out-patient), which aims to promote good health and alleviate ills and afflictions, is a significant contributor to climate change. Indeed, the carbon footprint of health care is equivalent to 4.4% of global net emissions.

**Fig. 1:**
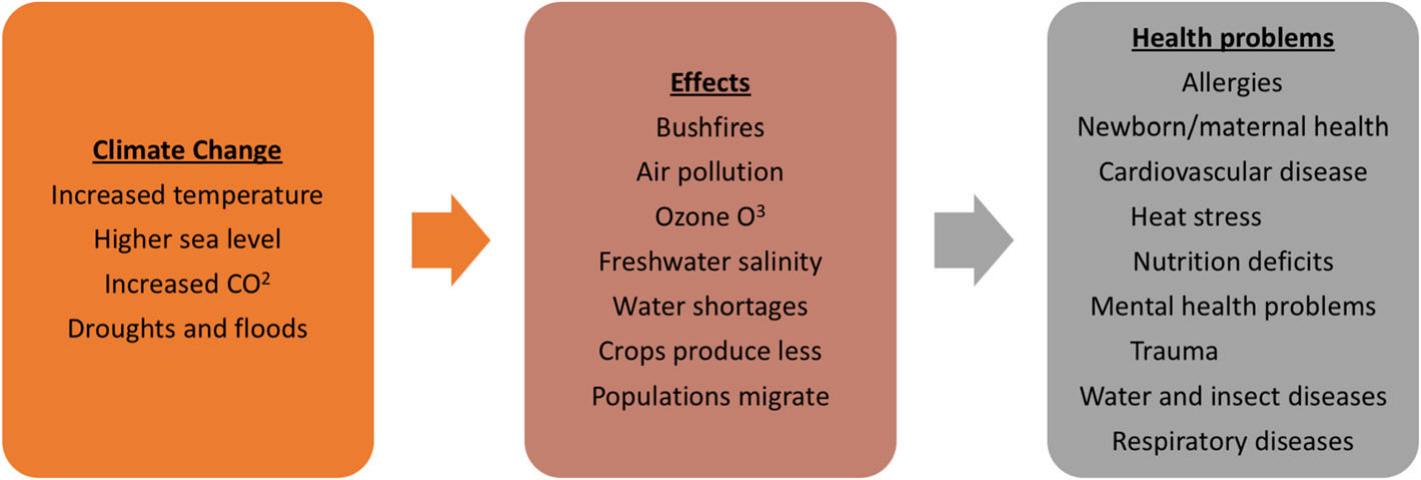
Schematic depicting the elements of climate change from increasing greenhouse gas emissions, the events and effects, and an overview of resulting health problems and diseases

Energy use is at the core of health care emissions (over half), and primarily emanate from fossil fuels. Total health care emissions can be sub-divided:

- those directly from health care facilities, 17%
- indirect emissions from heating/cooling, power purchases, 12%
- most are from the health care supply chain, ie the production, transport, use and disposal of goods/services, 71%. (Fossil fuels are central to greenhouse gas emissions [2] (Fig. 1).

*‘Primum non nocere’* [L: first do no harm] attributed to Hippocrates, guides clinicians to act for their patient’s *benefit*. Given this apparent dissonance, ‘Health Care Without Harm’, as an independent environmental engineering organisation, works to make the world’s health sector ecologically sustainable, by reducing waste, reducing toxic chemicals, transforming supply chains, and advocating for climate change [2].

‘Health Care without Harm’ as a principle, is what all medical and health practitioners should aspire to, and yet as a sector, we contribute to health harm, due to climate change from greenhouse gases and global warming.

## Climate change and health

The relationship between climate change and poor health has many components, but in essence, fossil fuel use increases atmospheric CO_2_ (also CH_4_, N_2_O), which causes higher temperatures and rising sea levels, which then induce floods and droughts [3]. Figure 1 illustrates the passage for poor health, which follows freshwater salinity, bush and forest fires, ozone damage, water shortages, air pollution, lower crop yields and less nutritional harvests, in turn, potentiating migration from unsustainable locale. The health effects and diseases are many, and disproportionately affect those inherently more vulnerable, ie children, older people, pregnant women, those with pre-existing morbidity (3).

In addition to health vulnerability, there is further exposure for communities with geographic and income disadvantage, eg Pacific Island nations. An example, familiar to me, is Bangladesh, where the Bay of Bengal tideline is moving northward, major flooding of the Brahmaputra River inundates villages, wipes out health clinics, and dirties clean water wells [3]. Together with increased periods of drought and reduced crop yields, millions of people are now migrating from rural farms to the capital city Dhaka, already bursting with pollution, and slums [3].

There is correlation between a country’s health care climate footprint and its Gross Domestic Product (GDP) health budget [2]. In Bangladesh, just 2.34% GDP is allocated to health care, and emissions are relatively low (similarly India), in comparison to high income countries. Table 1 provides examples of this disparity.

**Table 1:** Gross Domestic Product (GDP) spent on health care correlates with carbon dioxide emissions resulting from health care activity

	% GDP on health care	Health care footprint (tCO_2_e/capita)	Health care % of national footprint
USA	16.5	1.72	7.6
UK	9.7	0.66	5.4
Australia	9.1	1.29	5.1
India	3.6	0.03	1.5

(World Bank, 2018: https://data.worldbank.org/indicator/SH.XPD.CHEX.GD.ZS?locations=BD);

Arup https://www.arup.com/perspectives/publications/research/section/healthcares-climate-footprint

## Global overview

The Paris agreement, the United Nations sustainable developments goals (SDGs), and the WHO climate change goals intersect [1, 2]. All acknowledge that the main environmental impacts of health systems come from consumption of energy and resources, production of greenhouse gas emissions, use and disposal of toxic chemicals, and production of waste and wastewater. These impacts are associated with health-care facilities, but most take place *upstream* (ie before a product reaches a healthcare site), associated with procured goods and services [4].

The Australian Medical Association’s position statement, calls on health and medical professionals to:

- Lead on environmental sustainability by drawing it into focus at all levels, including by requiring consideration of an environmental sustainability impact statement in decision-making processes
- Educate themselves and their colleagues about the importance of environmental sustainability, and how practices and processes in their workplaces might become more sustainable
- Understand the synergy between improving healthcare environmental sustainability and improved healthcare effectiveness, efficiency, and financial sustainability
- Actively participate in environmentally sustainable practices in their workplaces and encourage behavioural change [5].

Similarly, the Australian Podiatry Association is gearing up to lead in this important space, by developing a strategic approach to educate members to adopt low-emission activity wherever possible. It is very clear, that the health sector, whose mission is protecting and promoting health, also contributes to the climate crisis — *the greatest health threat of the twenty-first century* — and therefore, has an important role to play in its alleviation.

## SDG 12, responsible consumption and production, how podiatrists can reduce emissions

The United Nations has 17 SDGs, signed by all United Nations Members and agreed to work towards achieving by 2030 [6]. The SDGs provide a vision to free the world from poverty, hunger and disease.

The main areas for Podiatry clinics to target are energy, product use and disposal, and especially the ‘upstream’ factors involved in the production, packaging, transporting and disposal of clinic stock and energy supply. Table 2 sets out some ideas to consider, and further options include:

- Telehealth, which kept me connected with patients during pandemic lock downs. Many GPs have continued telehealth permanently [5].
- Sustainable footwear is increasingly sought by consumers. *Green footwear* is a relatively new, rapidly evolving, and exciting area for podiatrists as patients demand more sustainable products. A summary list of *Green footwear* is available [7].
- Communicate and advocate for ‘big picture health’. Podiatrists are in a great position to educate, and empower people to attain recommended physical activity levels [8].
- WHO have physical activity guidelines for all ages [9].
- Parkrun is accessible, flexible (ie walk/ walk-run/ run) and builds all-age physical activity communities, encouraging people to move more. Time is green space is good for total health, and increasingly relevant as populations continue to urbanise.

**Table 2:** Green Podiatry actions require variable effort, and involve a range of cost: benefits

Effort estimates	Action examples
Easy, immediate	e-comms, e-records (data security!), reusable shopping bags, buy local, less packaging, turn off lights, adjust temperature settings re heat/cooling
Some organisation	LED lighting, avoid packaging as much as possible, and set up recycling eg paper /plastics/ foils; use e-waste and recycling depots
More commitment	Public transport/walk/ride a bicycle as possible (even 1–2 days/week to work – with a friend/colleague?), switch to renewable energy (add solar panels – work/home), car share commutes
Bigger issues	Limit air travel, esp. long haul overseas
	Plan for an e-car
	Purchase power (eg buy local)
	Contact your MP, think about your vote

## Did the CoV2 SARS pandemic mitigate climate change?

In his recent book, Bill Gates states that there are two numbers which sum up climate change – 51 billion and zero. This reflects the 51 billion tons of greenhouse gases the world adds to the atmosphere annually, and zero is the target required to stop global warming and the dreadful fall out effects of not doing so [10].

Global emissions did reduce in 2020, with a slowed economy, but only to approximately 48 billion tons of carbon (5% reduction). Whilst flying and driving slowed right down, energy use to heat and cool, light and connect our spaces, produce and grow and transport goods and people continued. Gates provides a clear plan for getting to zero emissions. With significant innovation, it appears achievable and hopeful, but not easily so, as he attests [10, 11].

## The way ahead

Footprints are very familiar to podiatrists, and evaluating your own carbon footprint may be helpful, in addressing your individual environment impact. There are many calculators available, and you can access one for your region, eg Australia [12].

Table 2 outlines some simple options. Clearly there are many more, and application will vary according to circumstances, motivation, and resources.

The *New York Times* [13], recently evoked a positive view of an imagined 2035*:*

- All of your electricity comes from wind, solar, and nuclear.
- Power plants are essentially gigantic batteries, slurping up renewable energy and storing it politely, emitting nary a belch of greenhouse gas.
- Your car is now electric. Charging your EV will take as long as filling your petrol car, and charging stations will have great coffee!
- Fewer extreme storms, wildfires, and droughts from eliminating fossil fuels.
- Less pollution also means fewer asthma attacks, strokes, and heart attacks.

## Conclusions

The Australian Podiatry Association is incorporating climate change into its strategic direction, with view to sharing this vision and the required actions with podiatrists, allied health, and medical colleagues globally.

As health professionals, our reach within the community is real, and our modelling of changed behaviour may be highly effective, as it was for smoking cessation 60 years ago [14, 15].

**Green Podiatry**, as a part of green healthcare, directs us to make lighter, cleaner footprints, as we traverse our beautiful world at this important time. Challenge, change, and hope will require careful balancing.

## Data Availability

N/A

## Abbreviations

CO_2_: Carbon dioxide
CH_4_: Methane
N_2_O: Nitrous oxide
GDP: Gross domestic product
SDGs: Sustainable development goals
WHO: World Health Organisation
UN: United Nations
CoV2 SARS: Coronavirus 2 Sudden Acute Respiratory Syndrome (Covid-19)
tCO_2_e/capita: tons of carbon dioxide emissions per country’s population (per head)
GP: General Practitioner (doctor)
MP: Member of Parliament
EV: Electric Vehicle
